# Cost of Living with Parkinson's Disease over 12 Months in Australia: A Prospective Cohort Study

**DOI:** 10.1155/2017/5932675

**Published:** 2017-03-02

**Authors:** Shalika Bohingamu Mudiyanselage, Jennifer J. Watts, Julie Abimanyi-Ochom, Lisa Lane, Anna T. Murphy, Meg E. Morris, Robert Iansek

**Affiliations:** ^1^Centre for Population Health Research, School of Health & Social Development, Faculty of Health, Deakin University, Burwood, VIC 3125, Australia; ^2^Clinical Research Centre for Movement Disorders and Gait, The National Parkinson Foundation Centre of Excellence, Kingston Centre, Monash Health, Cheltenham, VIC 3192, Australia; ^3^School of Clinical Sciences Monash University, Clayton, VIC 3168, Australia; ^4^Healthscope, Northpark Private Hospital, Plenty and Greenhills Roads, Bundoora, VIC 3083, Australia; ^5^School of Allied Health, La Trobe University, Bundoora, VIC 3083, Australia

## Abstract

*Background*. Parkinson disease (PD) is a costly chronic condition in terms of managing both motor and nonmotor symptoms. The burden of disease is high for individuals, caregivers, and the health system. The aim of this study is to estimate the annual cost of PD from the household, health system, and societal perspectives.* Methods*. A prospective cohort study of newly referred people with PD to a specialist PD clinic in Melbourne, Australia. Participants completed baseline and monthly health resource use questionnaires and Medicare data were collected over 12 months.* Results*. 87 patients completed the 12-month follow-up assessments. The mean annual cost per person to the health care system was $32,556 AUD. The burden to society was an additional $45,000 per annum per person with PD. The largest component of health system costs were for hospitalisation (69% of total costs). The costs for people with moderate to severe disease were almost 4 times those with mild PD ($63,569 versus $17,537 *p* < 0.001).* Conclusion*. PD is associated with significant costs to individuals and to society. Costs escalated with disease severity suggesting that the burden to society is likely to grow with the increasing disease prevalence that is associated with population ageing.

## 1. Introduction

Parkinson's disease (PD) is a chronic and degenerative neurological condition that is associated with lifelong disability [1]. Movement disorders such as slowness, balance impairment, tremor, freezing, and rigidity are characteristic of PD and nonmotor symptoms such as anxiety, depression, fatigue, and cognitive impairment are common [2]. Modified Hoehn and Yahr (HY) score is the clinical rating method to determine the level/severity of motor function in people with PD [3]. Progression in HY stages correlates with deterioration in an individual's quality of life [4, 5]. As the disease progresses, people with PD become highly vulnerable to falls and fall-related injuries [6]. The range of symptoms associated with PD means that the disease burden to the household (individual and family), health system, and society is usually significant.

Parkinson's disease is the second most common neurological disorder after Alzheimer's disease [1, 7]. The number of new cases of PD in Australia grew by 17% during the period 2005 to 2011 [8]. Average life expectancy after a diagnosis of PD is around 12 years although people can live more than 20 years with comprehensive care [9]. A European study of six countries highlighted that 160 in 100,000 people aged 65 years and older population have a diagnosis of PD and in future this will increase with rapid population aging [10].

A US study estimated that the annual health system cost of PD per person was in the range $1,750–$17,560 USD in 2002 [11] but in 2010 this was closer to $23,000 USD per person with the national burden of PD exceeding $14 billion USD [12]. In Australia the cost to the individual with PD was more than $15,000 per year in 2011 [8]. There was a significant burden to the health system ($8,000 per year in 2014 [8]) including hospitalisations and pharmaceutical and medical services. A recent prospective cohort study indicated that medication expenses contributed nearly 35% of total health system costs related to PD [13]. People with PD and their families also face significant out-of-pocket expenses for care-giving and loss of productivity [14].

A number of international and Australian studies have investigated the health-related quality of life (HRQOL) of people with PD [5, 15]. Several studies have evaluated the economic burden related to caring for people with PD [13, 16–18] and some have focused on the association between resource utilisation and disease severity [18].

Given the paucity of recent data on the costs of PD, the aim of this paper is to estimate the costs of PD over 12 months from the perspective of the household, the health system, and society. A secondary aim is to investigate the impact of disease severity on annual costs and resource use.

## 2. Methodology

### 2.1. Study Design

This was a prospective cohort study with a 12-month follow-up conducted in Melbourne, Australia.

### 2.2. Study Population

The target population were people with idiopathic PD who were newly referred to a specialist PD clinic in metropolitan Melbourne [19].* Inclusion criteria* for the study were (i) confirmed diagnosis of idiopathic PD; (ii) informed consent to participate in the study; (iii) ability to attend assessment clinics; and (iv) ability to complete questionnaires over 12 months.* Exclusion criteria* were (i) coexisting neurological conditions and (ii) disease category of HY stage five. Participants were also asked to consent to retrieval of their data from Medicare Australia, but nonconsent did not preclude them from participating in this study.

### 2.3. Ethics Approval

Ethics approvals were obtained from the Southern Health Human Research Ethics Committee (HREC number 06107B) and Monash University Standing Committee on Ethics in Research Involving Humans (SCERH number 2006/728MCC).

### 2.4. Data Collection

Participant's health services resource utilisation over 12 months was assessed through a series of questionnaires administered monthly, at baseline, 3 months, and 12 months. The baseline and 3- and 12-month questionnaires assessed PD duration and severity. Home based care services and community services (informal care, formal care, and meal-on-wheels) were also collected at 3 and 12 months. Participants were assessed by a trained assessor at home, or outpatient clinic [19]. Data related to health service resource use were collected via the monthly questionnaires [19]. These included questions to assess hospital admissions (length of stay, name of hospital, and method of transport to hospital), medical services (general practitioner, medical specialist, imaging services, and pathologist), and allied health services (physiotherapy, podiatry, etc.). Monthly questionnaires were completed with the help of a project officer who met with participants. Where participants consented, individual data on medical services and pharmaceutical use over 12 months were obtained from the national insurer, Medicare Australia. Resources were categorised according to the perspective of the analysis: individual/household, health system, or limited societal (inclusive of informal care but not productivity losses).  Table 1 shows the sources of data collection and perspective for cost estimates.

### 2.5. Cost Analysis

Costs were attributed to self-reported resource utilisation according to service category. Costs for Medicare Australia data were reported as the individual out-of-pocket component, government cost (benefit paid) and societal cost (out-of-pocket + government). All costs are reported in 2012 Australian dollars.

#### 2.5.1. Hospitalisation

Hospitalisation data including number of admissions and total length of stay (LOS) over a 12-month period for both private and public hospitals for all causes were obtained from self-report via the monthly questionnaires. To estimate the cost of hospitalisation per participant, the mean cost of a hospital admission per day was calculated using the national average cost per weighted separation from the Independent Hospital Pricing Authority (IHPA) [21] and the average hospital length of stay from the Australian Institute of Health and Welfare (AIHW) [20] according to the formulas shown in Table 2. Where resource data were missing from the monthly questionnaires, data for the number of hospital admissions and total length of stay over 12 months were imputed based on a weighted average from the available data for each participant.

#### 2.5.2. Medical Services and Pharmaceuticals

Medicare Australia data were obtained for consenting participants and used to determine the total charges and benefits paid for visits to general practitioners, medical specialists (including private hospital visits), pathology, imaging, and pharmaceuticals over 12 months. For participants who did not consent to Medicare data an imputation method was used to replace missing data based on disease severity according to HY score; less than 2.5 for mild disease and moderate to severe if HY was equal to or more than 2.5 [7].

#### 2.5.3. Allied Health Services

The costs of physiotherapy and podiatry services were estimated from both Medicare data and self-reported data taken from resource use questionnaires. Where both sources of data overlapped, preference was given to Medicare data. Costs for occupational therapy, speech therapy, psychology, dietetics, chiropractor, and optometry services were analysed using self-reported data. “Other services” included remedial massage and naturopathy services. Cost of allied health services was calculated from the number of visits and unit cost (Table 2) for each health service (for self-reported data).

#### 2.5.4. Other Medical Services

Cost of dental visits was analysed using unit costs from the Australian Government Department of Veterans Affairs and study data were gathered from self-reported data taken from resource use questionnaires.

#### 2.5.5. Home Based Care and Community Services

Formal care included both personal care assistants (PCA) and home based nursing care. PCA and nurses helped with showering, dressing, and regular review. The resource use questionnaire included data on how often home carers visited (twice daily, daily, every second day, and other). It was assumed the duration of each visit was 1 hour and the cost for home based nursing was $27 per hour [22] and for PCA was $25 per hour [22] (Table 2). “Other community services” included were the provision of meals by meal on wheels; the daily cost was assumed to be $16.50 [22]. The number of informal care hours per week was obtained from the self-reported questionnaires at 3 and 12 months. The total cost of informal care was estimated by multiplying total number of informal care hours over 12 months (multiplying weekly informal care hours by 52) by an hourly dollar value for informal care of $25 per hour [22] (Table 2).

## 3. Results

### 3.1. Participants

**Figure 1:**
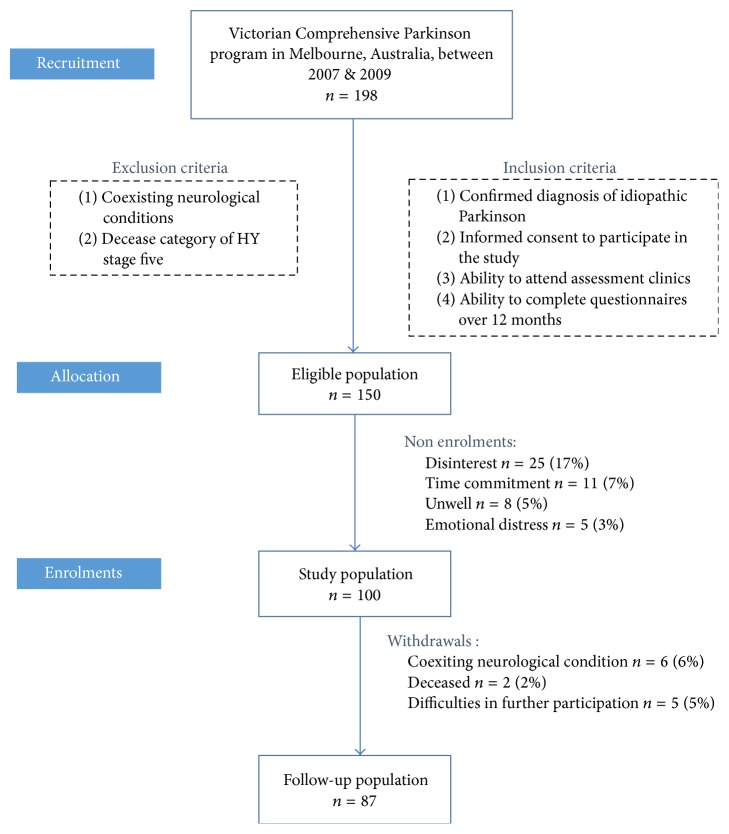
Consort chart; study population.

198 people with PD attended the Victorian Comprehensive Parkinson's Programme (VCPP) and, of this population, 150 people who met the inclusion criteria were invited to participate in the study (Figure 1). From this population 100 people were willing to participate in the study. From the sample population (*n* = 100) 13 participants withdrew over the 12-month period due to coexisting neurological conditions (*n* = 6) and deceased (*n* = 2) and difficulties in further participation (*n* = 5). There were 87 participants who completed the study at 12 months.

The age of the study population ranged from 43 to 89 years with a mean age of 69 years. 71% of the study population were aged 65 years and older (Table 3). There were 36 females (42%) and 51 males (58%) who participated in the study. There were 52 (60%) who had moderate to severe PD (HY equal or more than 2.5) and 35 (40%) with mild disease (HY less than 2.5). Most of the participants were in HY stage 2.5 (*n* = 26, 30%) while HY stage 1.5 (*n* = 2, 2%) had the least number of participants.

### 3.2. Resource Utilisation and Cost Analysis

#### 3.2.1. Hospitalisation

The study population had a mean number of hospital admissions per person of 1.01 (SD 1.31), with a mean total length of stay over 12 months of 7.1 (SD 9.11) days (Table 4). This differed by severity; for people with mild disease the mean annual number of days in hospital was 4 days compared to 20 days in the people with more severe PD (*p* < 0.001). Similarly the cost of all hospitalisations (public and private) over 12 months differed by severity with a mean annual cost of $6,160 (SD 9,292) in people with mild disease to $30,061 (SD 40,732) (*p* < 0.001) for those with more severe PD (Table 5). The mean cost of transport to hospital over 12 months including ambulance, taxi, or private vehicle was $362 (SD 799), of which the mean cost of ambulance was $338 (SD 791) per person. The single largest reason for hospital admissions was to optimize/adjust Parkinson medications (42%), a further 21% of hospitalisations were directly or indirectly related to PD (falls injuries, pain management, deep brain stimulation and surgical procedures), and 17% of hospitalisations were for reasons not related to PD or an unknown reason. Two participants reported an admission for deep brain stimulation treatments, each with a LOS of 22 days.

#### 3.2.2. Pharmaceuticals

The mean total cost of all prescribed medications was $3,644 (SD 2,240) for people with mild disease and $5,601 (SD 2,573) for more severe cases of PD over 12 months (*p* < 0.001) (Table 5). This comprised a mean benefit paid per person of $3,144 (SD 2,311) and $5,011 (SD 2,453) for mild and moderate to severe disease, respectively. Mean out-of-pocket charges were $490 (SD 195) for people with mild disease and $596 (SD 26) for people with more severe disease.

#### 3.2.3. Medical Services and Allied Health Services

There was no difference in the annual number of visits to a general practitioner for people with mild and moderate to severe disease; however, people with moderate to severe disease had more than twice the number of visits to a medical specialist compared to those with mild disease (28.3 versus 12; *p* < 0.001) (Table 4). The mean annual cost of visits to a medical specialist was $4,272 (SD 6,251) for people with moderate to severe disease and $2,706 (SD 2,429) for people with mild disease (*p* = 0.08). The mean annual out-of-pocket cost for all medical services was $1,179 (SD 1,113) for people with mild disease and $1,464 (SD 2,309) for people with moderate disease (*p* = 0.25) (Table 5).

In addition people with PD consulted a range of allied health practitioners over 12 months. For people with mild disease the mean total cost of all allied health was $564 of which $447 (79%) were for physiotherapy. For people with moderate to severe disease the total cost was $971 with $627 (65%) for physiotherapy and $148 (15%) for podiatry. For both groups 86% of allied health professional costs were paid out-of-pocket (Table 5).

#### 3.2.4. Home Based Care, Community Services, and Informal Care

The study population had an average of 2 hours of nurse visits per week and 4 hours per week of PCA visits with a mean cost of $121 (SD 698) for nursing care and $463 (1,598) for PCA care per person per year (Tables 4 and 5). It was only people with moderate to severe disease who reported the use of community-based nursing care (Table 4). Informal care contributed to the largest economic burden in the PD population. Fifty-two percent of the study population relied on informal care and for most of them a family member who lived with them helped with their daily activities. The mean number of hours was 24 hours per week from the primary informal caregiver (Table 4). In addition to this, people with PD reported an average of 3 hours of help from an additional carer each week. Other community-based services reported were the provision of meals on a regular basis. Fourteen percent of the study population reported using this service at a mean cost of $370 (SD 1,233) per person annually.

#### 3.2.5. Total Cost

**Figure 2:**
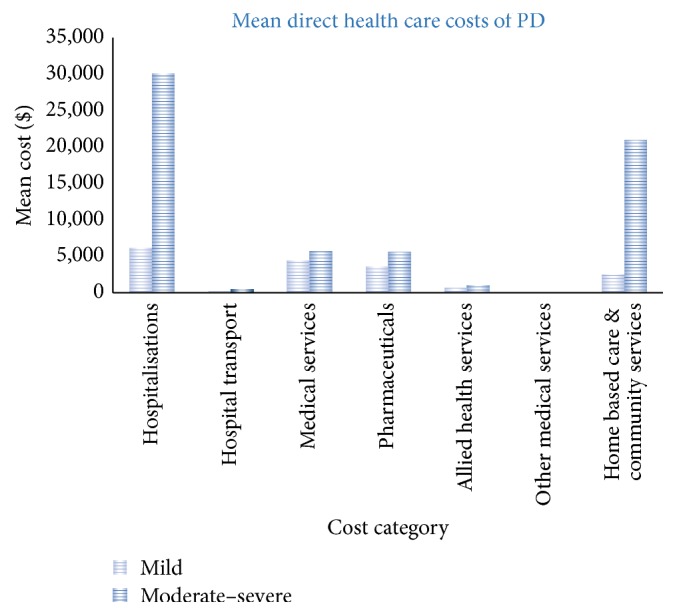
Cost of Parkinson's disease per year per person.

**Figure 3:**
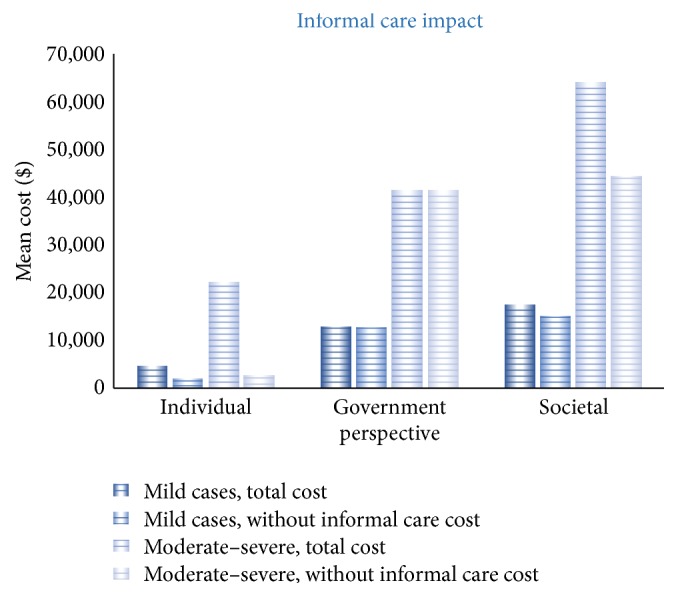
Cost impact of informal care.

Mean total cost per person from a societal perspective of living with PD over 12 months for the entire study population was $45,104 (SD 46,446) (Table 5). From this 66% of costs were attributed to the health system and 34% a burden to households and individuals. Mean total cost varies according to disease severity (Figure 2); for mild cases the mean total societal cost was $17,537 (SD 17,397) and for people with moderate to severe disease was $63,659 (SD 50,629). Informal care represented 28% of total costs and differed according to whether PD was mild (informal care 14%) or moderate to severe (informal care 30%) (Figures 2 and 3).

## 4. Discussion

The mean annual cost to the health system for this cohort of people with PD was $29,916 (SD 36,532) per person in 2012 AUD. In 2016 this is equivalent to $32,300 AUD or $24,600 USD. In addition annual out-of-pocket expenses were $15,137 (SD 30,546) per person. This is slightly higher than a European study that estimated that the total cost per person with PD was €11,153 per year in 2010 (2012 AUD $14,020). This represented €5,626 direct medical costs, €4,417 direct nonmedical cost, and €1,109 for indirect costs (2012 AUD comparison: $7,020; $5,552; and $1,496) [30]. This represents a significant burden to both individuals and households and to the health care system. In our study two-thirds of the burden to the health care system was related to hospitalisation, with medical services and pharmaceuticals a significant contributor to total costs. As disease severity increased, the burden to the health system was even greater at more than three times that for people with mild PD. The increase in costs as disease progresses is consistent with findings from other studies with one international study determining that the direct cost related to PD doubled when disease progressed from HY stages I to IV [12]. A European study found that a one-unit increase on the dyskinesia severity scale (Part IVa of the Unified Parkinson's Disease Rating Scale (UPDRS)) resulted in an additional mean total cost of €737 per patient over a 6-month period [31].

The study participants were admitted to both private and public hospitals during the study period. There was an average of one admission per person with PD over a 12-month period with an average annual length of stay of 13.5 days. This compares to the average number of admissions of 0.8 for the Australian population aged over 55 years in 2009/10 [32, 33]. The main reason reported for hospitalisation was to adjust PD medication since frequent changes in symptoms require alteration of drug dosage and frequency. Other reasons for admission were for secondary causes including falls (9%). Other studies have found a higher rate of admissions due to the complications of PD [8].

Although there was no difference in the number of GP medical services by disease severity, the number of specialist medical services for the moderate to severe group was more than double for people with mild disease. Other than ongoing management of disease symptoms, a contributing factor to this difference could be that medical specialist services provided in private hospitals as part of the inpatient admission are billed to the national insurer, Medicare Australia. Although the number of admissions are similar between the two groups, the moderate to severe groups have five times the number of days in hospital over 12 months compared to the participants with mild disease. People with moderate to severe PD are more likely to receive specialist doctor visits the more days they are in hospital.

Medication use in the management of PD is ongoing and costly. The cost of all medications was 11% of the total cost of PD over the study period. The total annual cost of medications in this cohort was $418,775, of which the government paid $370,616 (86% of the total) and the remainder formed out-of-pocket costs for people with PD. With disease progression, drug dosage and frequency are likely to increase [34] and people with PD may need to take other medications to control the side effects of the drugs (e.g., to relieve nausea and vomiting and gastric reflux).

Due to disability and progressively increasing mobility conditions associated with PD, people living with PD often require allied health services such as physiotherapy, podiatry, and speech therapy to manage their strength and motor symptoms and to improve their quality of life [9, 13, 15]. The mean number of physiotherapy services reported was just under 9 visits per person per year, which represents less than one service per month. Factors contributing to this relatively low number of reported services might be that physiotherapy services may be provided as part of the hospital episode and not reported separately. An alternative explanation is that allied health services are often not claimable through Medicare Australia; therefore people are likely to face high out-of-pocket charges over 12 months. In this study allied health visits represented 27% of the total annual costs of PD to the household, excluding the burden of informal care.

From the study population almost 60% had moderate or severe PD implying that they have disability that affects their daily living. Participants reported that they were regularly assisted by a family member, or friends as carers in their day to day life. The largest burden from the individual/household perspective was the cost of informal care, estimated at $12,548 per person over 12 months. This was based on the opportunity cost method for valuing informal care that is on the assumption that if a family member was not able to provide this care then it would be the cost of equivalent care provided by a nursing care attendant. People with moderate to severe disease reported an average of 775 informal care hours annually. Only 33% of study participants reported receiving carer payments from the Australian federal government for the informal care they provided. In addition to informal care, participants received home nursing care and other services such as meals. The mean total cost of home based community care services was $950 per person over 12 months. It has been reported that between 2005 and 2011 the cost of informal care for people with PD doubled ($5.4 million versus $11.2 million) [8]. This included income in a formal work environment foregone by carers [35]. The growth reflects the additional number of people with PD and higher average earnings in the workforce. A number of studies suggest that the largest component of household burden was due to providing informal care and the subsequent loss of earnings [12, 36, 37]. Studies highlight that family relationships can be affected in the early stage of disease and it is important to be referred early for home help and counselling and to PD support groups [38, 39].

The strength of this study is the detail provided in the resource use questionnaires about the range and costs of health and community services utilised by people with PD. Combining these data with Medicare and pharmaceutical use over 12 months and including the frequency of questionnaires have provided an accurate picture of the resources used by someone with PD from several perspectives. A limitation was that participants were referred to a specialist PD clinic and therefore may not be representative of the PD community. By separately analysing the cohort by disease severity it is likely that those with more severe disease are similar to the PD population with moderate to severe disease in terms of health care resource utilisation. We were not able to obtain case level hospital admission data; therefore, our cost estimates are based on an average day of stay in an Australian hospital, with no reference to reason for admission or comorbidities.

Our population cohort included two people who had had deep brain stimulation (DBS) so we were not able to comment on the increased costs likely to be associated with DBS [40]. As DBS and other device-aided therapies are likely to become more common therapy for people with PD it is expected that the direct costs of managing PD will be higher in the future. In addition an ageing population means that people with PD are likely to live longer with increased disease severity. The relationship that we have found between higher costs and advancing disease and others have found with increased disability (based on the UPDRS) [31] will be more apparent in future studies. We recommend that future studies should determine the relative contribution to costs of different movement disorders, such as dyskinesia, freezing, bradykinesia, and postural instability.

## 5. Conclusion

The annual costs to the health system for people with PD are high, with more than two-thirds attributed to hospitalisation. Individuals also face a high out-of-pocket burden for nonhospital related health services and households face a burden from providing informal care, which represents approximately 28% of total costs. The difference in total costs by disease severity suggests that the burden to society is likely to grow in the future with increasing prevalence of chronic diseases such as PD in an ageing population.

## Figures and Tables

**Table 1:** Economic analysis perspectives and data collection sources.

	Perspective			Date source		
	Individual	Health system	Societal	Questionnaire at 3 and 12 months	Monthly questionnaire	Medicare Australia
*Hospitalisations*						
Public hospital	—	*∗*	*∗*	—	*∗*	—
Private hospital	—	*∗*	*∗*	—	*∗*	—
*Hospital transport*						
Ambulance (road and air)	—	*∗*	*∗*	—	*∗*	—
Private car	*∗*	—	*∗*	—	*∗*	—
Taxi	*∗*	—	*∗*	—	*∗*	—
*Medical services*						
General practitioner	*∗*	*∗*	*∗*	—	*∗*	*∗*
Medical specialist	*∗*	*∗*	*∗*	—	*∗*	*∗*
Imaging	*∗*	*∗*	*∗*	—	*∗*	*∗*
Pathology	*∗*	*∗*	*∗*	—	*∗*	*∗*
*Pharmaceuticals*						
Medication	*∗*	*∗*	*∗*	—	*∗*	*∗*
*Allied health services*						
Physiotherapist	*∗*	*∗*	*∗*	—	*∗*	*∗*
Podiatrist	*∗*	*∗*	*∗*	—	*∗*	*∗*
Occupational therapy	*∗*	*∗*	*∗*	—	*∗*	—
Speech therapy	*∗*	*∗*	*∗*	—	*∗*	—
Psychology	*∗*	*∗*	*∗*	—	*∗*	—
Dietetic	*∗*	*∗*	*∗*	—	*∗*	—
Chiropractic	*∗*	*∗*	*∗*	—	*∗*	—
Optometry	*∗*	*∗*	*∗*	—	*∗*	—
Other	*∗*	*∗*	*∗*	—	*∗*	—
*Other medical services*						
Dental	*∗*	*∗*	*∗*	—	*∗*	—
*Home based care and community services*						
Formal care, nurse	—	*∗*	*∗*	*∗*	—	—
Formal care, PCA	—	*∗*	*∗*	*∗*	—	—
Informal care	*∗*	—	*∗*	*∗*	—	—
Meals on wheels	—	*∗*	*∗*	*∗*	—	—

**Table 2:** Unit costs and assumptions for cost analysis.

Parameter	Unit cost ($)	Unit	Assumption/estimations	Data source

*Hospitalisations*				
Public hospital	1627.12	Per day	$\frac{NACWS*}{ALOS**}$	Australian hospital statistics 2011-12 [20] Independent Hospital Pricing Authority [21]
Private hospital	1334.24	Per day	$\frac{NACWS}{ALOS}\times 0.82$ Private hospital costs are weighted at 82% of public hospital costs to account for doctors charges which are included in Medicare data for private patients	Australian hospital statistics 2011-12 [20] Independent Hospital Pricing Authority [21]
*Hospital transport*				
Ambulance (road)	688.50	Per transport	Same average cost for both metropolitan and rural/remote region	Watts et al. (2013) [22]
Ambulance (air)	2682.30	Per transport	—	Victoria state government- Health [23]
Private car	0.66	Per kilometre	Average of 30 km for full journey and same average cost for all types of motor vehicles	Australian government taxation office- car expenses [24]
Taxi	5.54	Per kilometre	Average of 30 km for full journey	Taxi service commission [25]
*Allied health services*				
Physiotherapy	62.25	Per visit	85% covered by Medicare Out-of-pocket cost is $9.30	MBS item 10960 [26]
Podiatry	62.25	Per visit	85% covered by Medicare Out-of-pocket cost is $9.30	MBS item 10962 [26]
Occupational therapy	62.25	Per visit	85% covered by Medicare Out-of-pocket cost is $9.30	MBS item 10958 [26]
Speech therapy	62.25	Per visit	85% covered by Medicare Out-of-pocket cost is $9.30	MBS item 10970 [26]
Psychology	62.25	Per visit	85% covered by Medicare Out-of-pocket cost is $9.30	MBS item 10968 [26]
Dietetics	62.25	Per visit	85% covered by Medicare Out-of-pocket cost is $9.30	MBS item 10954 [26]
Chiropractic	62.25	Per visit	85% covered by Medicare Out-of-pocket cost is $9.30	MBS item 10964 [26]
Optometry	66.80	Per visit	For initial consultation	MBS item 10905 [26]
Remedial massage	55.92	Per visit	For initial consultation Total out-of-pocket cost	Worksafe Victoria item M600 [27]
Naturopathy	31.80	Per visit	Standard consultation Total out-of-pocket cost	Worksafe Victoria item N602 [28]
*Other medical services*				
Dental	53.55	Per visit	For a comprehensive oral examination Total out-of-pocket cost	Australian Government Department of Veterans Affairs-item D011 [29]
*Home based care and community services*				
Formal care, nurse	27.00	Per hour	Duration of formal nursing visit is equal to one hour	Watts et al. (2013) [22]
Formal care, PCA	25.00	Per hour	Duration of formal nursing visit is equal to one hour	Watts et al. (2013) [22]
Informal care	25.00	Per hour	—	Watts et al. (2013) [22]
Meals on wheels	16.50	Per day	Cost of meals per day is $16 for 2 meals and $16.50 for 3 meals	Watts et al. (2013) [22]

^*∗*^NACWS = national average cost per weighted separation [20]

^*∗∗*^ALOS = average length of stay [21].

**Table 3:** Population demographics.

	Mild	Moderate–severe	Study population
	*n* = 35	*n* = 52	
*Mean age (years)*	*68.1*	*69.6*	*69.0*
	*SD 9.4*	*SD 9.7*	*SD 9.6*
40–65 years (*n*)	12 (34%)	13 (25%)	25 (29%)
65+ years (*n*)	23 (66%)	39 (75%)	62 (71%)
*Gender*			
Female (*n*)	13 (37%)	23 (44%)	36 (42%)
Male (*n*)	22 (63%)	29 (56%)	51 (58%)
*Mini mental state examination (mean)*	*28.7*	*26.7*	*27.5*
	*SD 1.9*	*SD 4.0*	*SD 3.4*
*Disease duration (years)*	*2.7*	*8.2*	*6.0*
	*SD 3.1*	*SD 5.4*	*SD 5.3*
*Disease severity (HY stage) *	*35 (40.2%)*	*52 (59.8%)*	*87 (100%)*
Stage 1	22 (25.3%)	0	22 (25.3%)
Stage 1.5	2 (2.3%)	0	2 (2.3%)
Stage 2	11 (12.6%)	0	11 (12.6%)
Stage 2.5	0	26 (29.9%)	26 (29.9%)
Stage 3	0	16 (18.4%)	16 (18.4%)
Stage 4	0	10 (11.5%)	10 (11.5%)

**Table 4:** Resource utilization related to Parkinson's disease over 12 months.

	Mild *n* = 35	Moderate–severe *n* = 52	*p* value
*Hospitalisations*			
*Mean number of hospital admissions per person per year*	*0.60*	*1.29*	*<0.001*
	*SD 0.81*	*SD 1.29*	
Public hospital	0.11	0.62	0.01
	SD 0.32	SD 1.19	
Private hospital	0.49	0.67	0.20
	SD 0.74	SD 1.12	
*Mean hospital LOS (days) per person per year*	*4.43*	*20.15*	*<0.001*
	*SD 6.77*	*SD 26.20*	
Public hospital LOS	0.86	10.83	<0.001
	SD 2.81	SD 24.06	
Private hospital LOS	3.57	9.33	0.02
	SD 6.56	SD 16.58	
*Hospital transport (mean number times)*			
Ambulance (road + air)	0.11	0.58	0.01
	SD 0.32	SD 1.18	
Private car	0.43	0.98	0.02
	SD 0.65	SD 1.39	
Taxi	0.09	0.04	0.22
	SD 0.37	SD 0.19	
*Medical services (mean number of visits)*			
General practice	10.17	10.25	0.47
	SD 4.62	SD 4.35	
Medical specialist Services	12.00	28.32	<0.001
	SD 9.66	SD 18.56	
Imaging	4.50	2.98	<0.001
	SD 1.90	SD 1.53	
Pathology	13.51	18.58	0.05
	SD 5.70	SD 16.92	
*Allied health services (mean number of visits)*			
Physiotherapy	7.11	10.06	0.15
	SD 12.66	SD 12.99	
Podiatry	1.06	2.38	0.01
	SD 1.86	SD 3.09	
Occupational therapy	0.34	0.83	0.06
	SD 0.76	SD 1.69	
Speech therapy	0.03	0.56	0.04
	SD 0.17	SD 1.70	
Psychology	0.03	0.30	0.04
	SD 0.17	SD 0.82	
Dietetics	0.00	0.21	0.12
	SD 0.00	SD 1.07	
Chiropractic	0.03	0.44	0.09
	SD 0.17	SD 1.81	
Optometry	0.06	0.04	0.34
	SD 0.24	SD 0.20	
Other	1	1.02	0.49
	SD 1.96	SD 2.57	
*Other medical services (dental) (mean number of visits)*	1.11	0.25	0.08
	SD 4.15	SD 1.40	
*Home based care and community services (mean number of hours)*			
Formal care hours, nurse	—	7.00	—
		SD 30.94	
Formal care hours, PCA	—	31.00	—
		SD 80.60	
Informal care hours	96.2	775	0.01
	SD 354.53	SD 1492.37	
Meals on wheels	11.89	89.00	0.05
	SD 50.58	263.41	

**Table 5:** Mean annual cost per person with PD from individual, health system, and societal perspectives ($AUD).

Cost perspective	Mild *n* = 35			Moderate–severe *n* = 52			Total study population *n* = 87		
	Individual ($)	Health system ($)	Societal ($)	Individual ($)	Health system ($)	Societal ($)	Individual ($)	Health system ($)	Societal ($)
*Hospitalisations *	*00*	*6,160*	*6,160*	*00*	*30,061*	*30,061*	*00*	*20,446*	*20,446*
	*SD 00*	*SD 9,292*	*SD 9,292*	*SD 00*	*SD 40,732*	*SD 40,732*	*SD 00*	*SD 34,014*	*SD 34,014*
Public hospital	00	1,395	1,395	00	17,617	17,617	00	11,091	11,091
	SD 00	SD 4,571	SD 4,571	SD 00	SD 39,153	SD 39,153	SD 00	SD 31,326	SD 31,326
Private hospital	00	4,765	4,765	00	12,444	12,444	00	9,355	9,355
	SD 00	SD 8,751	SD 8,751	SD 00	SD 22,117	SD 22,117	SD 00	SD 18,294	SD 18,294
*Hospital transport *	*23*	*193*	*215*	*26*	*436*	*461*	*25*	*338*	*362*
	*SD 61*	*SD 643*	*SD 643*	*SD 41*	*SD 870*	*SD 881*	*SD 50*	*SD 791*	*SD 799*
Ambulance (road + air)	00	193	193	00	436	436	00	338	338
	SD 00	SD 943	SD 943	SD 00	SD 870	SD 870	SD 00	SD 791	SD 791
Private car	8	00	8	19	00	19	15	00	15
	SD 13	SD 00	SD 13	SD 28	SD 00	SD 28	SD 23	SD 00	SD 23
Taxi	14	00	14	6	00	6	10	00	10
	SD 62	SD 00	SD 62	SD 32	SD 00	SD 32	SD 46	SD 00	SD 46
*Medical services *	*1,179*	*3,217*	*4,394*	*1,464*	*4,194*	*5,664*	*1,349*	*3,793*	*5,141*
	*SD 1,113*	*SD 1,533*	*SD 2,491*	*SD 2,309*	*SD 4,271*	*SD 6,470*	*SD 1,916*	*SD 3,460*	*SD 5,258*
General practitioner	40	430	469	41	371	411	41	395	434
	SD 63	SD 288	SD 293	SD 64	SD 397	SD 419	SD 63	SD 357	SD 372
Medical specialist	929	1,777	2,706	1,229	3,043	4,272	1,108	2,534	2,883
	SD 1,020	SD 1,565	SD 2,429	SD 2,281	SD 4,088	SD 6,251	SD 1,876	SD 3,357	SD 3,593
Imaging	144	672	815	133	469	602	137	551	688
	SD 132	SD 334	SD 413	SD 186	SD 271	SD 435	SD 166	SD 312	SD 437
Pathology	67	338	404	60	298	359	63	314	377
	SD 80	SD 251	SD 315	SD 90	SD 204	SD 262	SD 86	SD 224	SD 284
*Pharmaceuticals *	*490*	*3,144*	*3,644*	*596*	*5,011*	*5,601*	*554*	*4,260*	*4,814*
	*SD 195*	*SD 2,311*	*SD 2,240*	*SD 26*	*SD 2,453*	*SD 2,573*	*SD 241*	*SD 2,555*	*SD 2,616*
*Total allied health *	*544*	*56*	*564*	*802*	*171*	*971*	*698*	*125*	*807*
	*SD 824*	*SD 86*	*SD 833*	*SD 938*	*SD 245*	*SD 1,045*	*SD 898*	*SD 190*	*SD 981*
*Dental services *	*60*	*0.00*	*60*	*13*	*0.00*	*13*	*32*	*0.00*	*32*
	*SD 222*	*00*	*SD 222*	*SD 75*	*00*	*SD 75*	*SD 153*	*00*	*SD 153*
*Home based care and community services*	*2,405*	*95*	*2,500*	*19,375*	*1,532*	*20,908*	*12,548*	*954*	*13,502*
	*SD 8,863*	*SD 405*	*SD 8,846*	*SD 37,309*	*SD 3,024*	*SD 37,024*	*SD 30,440*	*SD 2,448*	*SD 30,434*
Formal care, nurse	00	00	00	00	775	775	00	463	463
	SD 00	SD 00	SD 00	SD 00	SD 2,015	SD 2,015	SD 00	SD 1,598	SD 1,598
Formal care, PCA	00	00	00	00	203	203	00	121	121
	SD 00	SD 00	SD 00	SD 00	SD 887	SD 887	SD 00	SD 698	SD 698
Informal care	2,405	00	2,405	19,375	00	19,375	12,548	00	12,548
	SD 8,863	SD 00	SD 8,863	SD 37,309	SD 00	SD 37,309	SD 30,440	SD 00	SD 30,440
Meals on wheels	00	95	95	00	555	555	00	370	370
	SD 00	SD 405	SD 405	SD 00	SD 1,509	SD 1,509	SD 00	SD 1,211	SD 1,211
*Mean total cost*	*4,701*	*12,865*	*17,537*	*22,277*	*41,396*	*63,659*	*15,137*	*29,916*	*45,104*
	*SD 9,429*	*SD 10,917*	*SD 17,397*	*SD 37,221*	*SD 42,862*	*SD 50,629*	*SD 30,546*	*SD 36,532*	*SD 46,446*
*Mean total cost without informal care*	*2,296*	*12,865*	*15,132*	*2,902*	*41,396*	*44,284*	*2,589*	*29,916*	*32,556*
	*SD 1,475*	*SD 10,917*	*SD 10,909*	*SD 2,530*	*SD 42,862*	*SD 42,794*	*SD 2,184*	*SD 36,532*	*SD 36,603*
